# Maternal Cigarette Smoke Exposure Exaggerates the Behavioral Defects and Neuronal Loss Caused by Hypoxic-Ischemic Brain Injury in Female Offspring

**DOI:** 10.3389/fncel.2022.818536

**Published:** 2022-02-18

**Authors:** Taida Huang, Xiaomin Huang, Hui Li, Junhua Qi, Nan Wang, Yi Xu, Yunxin Zeng, Xuewen Xiao, Ruide Liu, Yik Lung Chan, Brian G. Oliver, Chenju Yi, Dan Li, Hui Chen

**Affiliations:** ^1^Department of Pathology, The First Affiliated Hospital of Gannan Medical University, Ganzhou, China; ^2^Research Center, The Seventh Affiliated Hospital of Sun Yat-sen University, Shenzhen, China; ^3^School of Life Sciences, Faculty of Science, University of Technology Sydney, Sydney, NSW, Australia; ^4^Respiratory Cellular and Molecular Biology, Woolcock Institute of Medical Research, The University of Sydney, Sydney, NSW, Australia

**Keywords:** short-term memory, maternal SE, oxidative stress, motor function, inflammation

## Abstract

**Objective:**

Hypoxic-ischemic encephalopathy affects ∼6 in 1,000 preterm neonates, leading to significant neurological sequela (e.g., cognitive deficits and cerebral palsy). Maternal smoke exposure (SE) is one of the common causes of neurological disorders; however, female offspring seems to be less affected than males in our previous study. We also showed that maternal SE exaggerated neurological disorders caused by neonatal hypoxic-ischemic brain injury in adolescent male offspring. Here, we aimed to examine whether female littermates of these males are protected from such insult.

**Methods:**

BALB/c dams were exposed to cigarette smoke generated from 2 cigarettes twice daily for 6 weeks before mating, during gestation and lactation. To induce hypoxic-ischemic brain injury, half of the pups from each litter underwent left carotid artery occlusion, followed by exposure to 8% oxygen (92% nitrogen) at postnatal day (P) 10. Behavioral tests were performed at P40–44, and brain tissues were collected at P45.

**Results:**

Maternal SE worsened the defects in short-term memory and motor function in females with hypoxic-ischemic injury; however, reduced anxiety due to injury was observed in the control offspring, but not the SE offspring. Both hypoxic-ischemic injury and maternal SE caused significant loss of neuronal cells and synaptic proteins, along with increased oxidative stress and inflammatory responses.

**Conclusion:**

Oxidative stress and inflammatory response due to maternal SE may be the mechanism of worsened neurological outcomes by hypoxic-ischemic brain injury in females, which was similar to their male littermates shown in our previous study.

## Introduction

Neonatal Hypoxia-ischemic (HI) encephalopathy (HIE) is a common brain injury of neonates, which occurs in 3 per 1,000 term newborns and 6 per 1,000 preterm newborns (≤36 weeks of gestation) (Hagberg et al., 2015; Herrera et al., 2018). During the occurrence of hypoxic-ischemic injury, there is a significant decrease in blood oxygen delivery and blood flow to the affected brain areas, interrupting neonatal brain development which is the likely cause of neurological disorders associated with HI brain damage (Yong et al., 2012). The severity of HIE varies. Almost 40% of newborns with HIE were unable to survive the neonatal period, and 30% will suffer from some serious long-term neurological disorders, such as motor incoordination, cerebral palsy, visual impairment, seizures, epilepsy, intellectual disability, and learning disabilities (Higgins et al., 2011; Matsumoto et al., 2015; Li et al., 2017). Thus, perinatal HI brain injury brings major burdens to the families affected and the society.

Maternal cigarette smoke exposure (SE) is a well-accepted risk factor for fetal hypoxia, by decreasing the blood flow to the placenta due to the vasoconstriction effects of nicotine and reducing oxygen carrying capacity of both fetal and maternal red blood cells due to increased caroxyhemoglobin (Bureau et al., 1983; Philipp et al., 1984; Habek et al., 2002; Sen et al., 2010). In addition, *in utero* exposure to cigarette smoke or pure nicotine has been shown to cause motor, sensory and cognitive dysfunctions in the offspring from childhood, increasing the risk for attention-deficiency hyperactivity disorder and other neurobehavioral disorders (Batty et al., 2006; Julvez et al., 2007; He et al., 2017; Sifat et al., 2020). While sex difference has not been a focus in most human studies, one study suggests the odds seem to be more prevalent in boys (Minatoya et al., 2019). Our well-established mouse module of maternal SE during pregnancy replicated such neurological disorders in young male offspring (Chan et al., 2017b). However, another study in an Australian cohort failed to find any sex difference in such susceptibility (Silva et al., 2014).

Nevertheless, it is not surprising to observe that maternal SE during pregnancy can worsen the brain injury and neurological outcomes in adolescent offspring if the neonatal brains suffer from HI injury (Fren et al., 2002; Chan et al., 2017b). During the primary energy failure phase of HI, the lack of blood oxygen supply skews brain cells to anaerobic ATP synthesis with increased lactate production. The low ATP levels result in the failure of a series of mechanisms essential for the maintenance of cell integrity. For example, the sodium/potassium pumps dysfunction and increased intracellular calcium lead to cell necrosis and/or apoptosis in the affected brain region (Bano et al., 2017). The secondary energy failure phase occurs 6–48 h after the primary insults by HI, during which the accumulated lactate due to anaerobic ATP metabolism directly increases reactive oxygen species (ROS) levels, when the inflammatory response was activated by oxidative stress and chemicals released from the nearby dead neurons (Li et al., 2017). In this phase, oxidative stress and inflammation lead to further neuronal death predominantly through apoptosis, leading to neuronal loss in the injured brain and thereafter, neurological dysfunctions (Silveira and Procianoy, 2015; Lancelot et al., 2017). In our previous study, a sex difference in response to maternal SE was found, where increased brain oxidative stress was observed in male offspring but not the females (Chan et al., 2017a). This may be due to the better capacity of females to produce endogenous antioxidants (Chan et al., 2016b). As such, males from SE dams displayed worsened neurological defects after HI injury compared with those from the control dams. On the other hand, it raised the question of whether females from SE dams will have similar or better neurological outcomes after HI injury to those from the control dams.

Indeed, sexual difference is commonly observed in early life neurological conditions, such as higher incidence of Sudden Infant Death Syndrome (SIDS) in males (Fleming et al., 2015; Loomes et al., 2017). Clear evidence has shown a sexual dimorphism in neonatal HI caused cell death, brain lesion, and resulting behavioral and cognitive dysfunctions, in which mitochondrial dysfunction has been suggested to be the key player (Dukhande et al., 2009; Weis et al., 2012; Netto et al., 2017). The oxidative phosphorylation complexes in the mitochondrial electron transport chain are generally more active in females than males (Weis et al., 2012). When subjected to brain HI, the activity of antioxidant enzymes, such as glutathione, is increased in females, an adaptive and also potentially protective response which is absent in males (Demarest et al., 2016). This successful adaptive response in female offspring’s brains may also protect them from more severe functional damage in the event of a HI injury.

Therefore, we hypothesized that compared with those from control dams, female offspring from SE dams may have similar or even less severe neurological disorders as a result of HI brain injury. In this study, we used the female littermates from our previously published cohort of male mice that underwent HI injury at postnatal day (P) 10 (Chan et al., 2017b). We aimed to examine the changes in their neurocognitive and motor functions, as well as neural damage, inflammatory response, and oxidative stress in their brains at a young age.

## Materials and Methods

### Animal Experiments

The animal experiments were approved by the Animal Care and Ethics Committee at the University of Technology Sydney (ACEC# 2014-029). All procedures were performed according to the Australian National Health and Medical Research Council Guide for the Care and Use of Laboratory Animals.

The animal experiment has been published previously, and this study examined the female offspring from the same breeders in our previous study on their male littermates (Chan et al., 2017b). Briefly, female Balb/C mice (6-week-age, Australian Resource Centre, WA, Australia) were housed at 20 ± 2°C and maintained on a 12:12 h light/dark cycle (lights on 6:00 am) with *ad libitum* access to standard rodent chow and water. The dams (SE group) were exposed to cigarette smoke produced by two cigarettes (Winfield Red, ≤ 16 mg tar, ≤ 1.2 mg nicotine, and ≤ 15 mg of CO; VIC, Australia) inside a perspex box (19 L) in the fume hood, twice a day for 6 weeks before mating and during the gestation and lactation; the other half of the dams (SH group) were exposed to room air in an identical chamber at the same time. The whole body exposure allowed free movement and socialization during exposure. Male breeders and offspring were not exposed and remained in the home cage when the breeders were exposed. This protocol reflects babies from light smoking mothers (Benowitz et al., 2009; Vivekanandarajah et al., 2016). At P10, half of the female pups randomly selected from each litter were anesthetized with 2.5% isoflurane (1% O_2_, Veterinary Companies of Australia, NSW, Australia) and underwent left carotid artery occlusion by ligation as previously published (Chan et al., 2017b). The sham surgery was performed without carotid artery ligation. The wound was closed by Vetbond™ glue (3M, MN, United States). One hour after surgery, the pups were exposed to 8% oxygen (92% nitrogen) in a humidified chamber for 30 min in a 37°C water bath to induce HI injury. The pups with sham surgery were exposed to room air under the same conditions. This yielded four experimental groups, SH: offspring from sham exposed dam with sham surgery; HI: offspring from sham exposed dam with HI injury; SE: offspring from SE dam with sham surgery; HI + SE: offspring from SE dam with HI injury (*n* = 12).

The female offspring were sacrificed at P45 for brain tissue collection. After deep anesthesia with 2.5% isoflurane, mice were first transcardially perfused with 0.01 M phosphate-buffered saline (PBS) followed by 4% (w/v) paraformaldehyde in PBS. Then, the brains were incubated in 4% PFA in PBS at 4°C overnight. One the next day, the brain tissues were rinsed in PBS before the paraffin embedding procedure.

### Behavioral Tests

Behavioral tests were performed on the female offspring from P40 to 44, equivalent to the adolescent age in humans (Dutta and Sengupta, 2016). Animals were acclimated to the house with behavioral test apparatus for 3 days before the test phase, following our published protocols in their male littermates (Chan et al., 2017b).

#### Novel Object Recognition Test

To test the ability of short-term memory retention, each mouse was placed in a dark-colored box with two objects for two 5-min sessions (Chan et al., 2017b). In the first phase, two identical green square-shaped objects were placed for the familiarization session, and one of the objects was replaced with an orange triangular shaped object in the test session. The results were calculated as the percentage of time spent on the novel object over the total time spent on both objects in the testing phase. It is the nature of mice to explore a novel object over a familiar one; the mouse with memory deficits will not be able to identify the new object, therefore, will spend a similar period of time with each object, i.e., 50% on both objects.

#### Grip Traction Test

To test the muscle strength of the forelimbs, a grip traction test was performed by hanging the mouse onto a plastic rod (0.5 cm in diameter) by the forelimbs as previously published (Jones et al., 2008). The time was recorded from when the forelimbs were placed onto the rod until the mouse fell off onto a foam pad (50 cm below the rod).

#### Foot Fault Test

To assess the motor function, the mouse was placed on an elevated horizontal grid (size: 20 cm × 20 cm; square hole: 1 cm × 1 cm). The number of misplaced foot faults into the grid squares and the total number of footsteps within 2 min were recorded. These results were shown as the percentage of misplaced foot faults over the total number of steps during the test.

#### Elevated Plus Maze Test

To determine the anxiety level, elevated plus maze test was performed as previously described (Walf and Frye, 2007). The mouse was placed in the cross section facing the same open arm of the maze and recorded for 10 min. if a mouse is anxious, it will spend less time in the open arms. The results were shown as the percentage of time spent in the open arms over the 10 min.

### Immunohistochemistry

Immunofluorescence staining was assessed in the paraformaldehyde fixed paraffin embedded sections. Fixed brain tissue was dehydrated, cleared and infiltrated with paraffin wax by Tissue Processor™ (Thermo Fisher Scientific, MA, United States). Before immunofluorescence labeling, the sections were placed in xylene, and then hydrated through gradient ethanol with reduced concentrations to distilled water. Antigen retrieval was performed by immersing all slides in 10 mM sodium citric buffer (pH = 6.0) at 95°C for 45 min. The immunofluorescence labeling was performed using our previously published methods (Shang et al., 2020). The antibodies were diluted as per manufacturers’ recommendations neuron-specific class III beta-tubulin (Tuj1): 1:300, BioLegend, CA, United States, Cat#845502; HuC/D: 1:300, Invitrogen, MA, United States, Cat#A21271; Extracellular Leucine Rich Repeat And Fibronectin Type III Domain Containing 2 (ELFN2): 1:50, BIOSS, MA, Cat#bs7809-R; postsynaptic density protein 95 (PSD95): 1:200, GenTex, MI, United States, Cat#GTX133091; S100 calcium-binding protein B (S100b): 1:300, BD Biosciences, CA, United States, Cat#BD612376; Glial fibrillary acidic protein (GFAP): 1:300, Sigma-Aldrich, MO, United States, Cat#MAB1273; ionized calcium binding adaptor molecule 1 (Iba1): 1:300, Wako, Osaka, Japan, Cat#019-19741; interleukin 1 beta (IL-1β): 1:50, R&D Systems, MN, United States, Cat#MAB601; interleukin 6 (IL-6): 1:50 R&D systems, MN, United States, Cat#MAB401; Tumor necrosis factor α (TNFα): 1:50, R&D systems, MN, United States, Cat#MAB410; copper-zinc superoxide dismutase (SOD-1): 1:500, Santa Cruz Biotechnology, CA, United States, Cat#sc-101523; 4-hydroxynonenal (4-HNE): 1:100, NOVUS, MA, United States, Cat#NB100-63093. For terminal deoxynucleotidyl transferase dUTP nick end labeling (TUNEL), ApoAlert™ DNA Fragmentation Assay Kit was used following the manufacturer’s protocol (Clontech, CA, United States, Cat#630108). After staining, slides were mounted with a FluorSave™ anti-fading reagent (Millipore, MA, United States, Cat#345789) containing 1.5 μg/ml 4′, 6′-diamidino-2-phenylindole hydrochloride (DAPI, Sigma-Aldrich, MO, United States, Cat#268298). The region of primary/secondary motor cortex and dentate gyrus region of the hippocampus were imaged by a confocal microscope (ZEISS-LSM800, Oberkochen, Germany).

### Statistical Analysis

Four images from the serial coronal sections of each brain were randomly selected for each analysis using Image J (Schneider et al., 2012). The percentage of immunoreactive cells was normalized by the number of DAPI staining in the same area, and relative immunofluorescence intensity was normalized by the average intensity of SH group in each bar chart. The results are presented as mean ± SEM, and the differences were analyzed by two-way ANOVA followed by *post hoc* Turkey tests (Prism 8.0, GraphPad Software, CA, United States). *P* < 0.05 was considered statistically significant.

## Results

### Maternal Smoke Exposure Slowed Down Postnatal Growth

SE offspring had significantly smaller body and brain weights at P45 (*P* < 0.05, body weight: *F* = 12.34 and brain weight: *F* = 10.98, SE vs. SH, Table 1). However, the percentage of brain weight was not different between SH and SE groups (Table 1). HI injury at P10 did not affect the body weight, nor brain weight of adolescent offspring from either control of SE dams (Table 1).

**TABLE 1:** Anthropometric parameters of the female offspring at P45.

Female offspring	SH *n* = 12	HI *n* = 12	SE *n* = 12	HI + SE *n* = 12
Body weight (g)	17.0 ± 0.32	16.1 ± 0.34	15.2 ± 0.31*	16.0 ± 0.38
Brain weight (g)	0.299 ± 0.003	0.298 ± 0.003	0.285 ± 0.003*	0.292 ± 0.003
Brain%	1.78 ± 0.02	1.80 ± 0.04	1.88 ± 0.03	1.83 ± 0.04

The results are expressed as mean ± SEM. *P < 0.05, compared with the SH offspring. SH, from sham exposed dams with sham surgery; HI, hypoxic-ischemic injury; SE, from smoke exposed dams with sham surgery; HI + SE, from smoke exposed dams with hypoxic-ischemic injury.

### Maternal Smoke Exposure Worsened the Deficits in Short-Term Memory and Motor Functions Due to Hypoxia-Ischemic Injury

**FIGURE 1:**
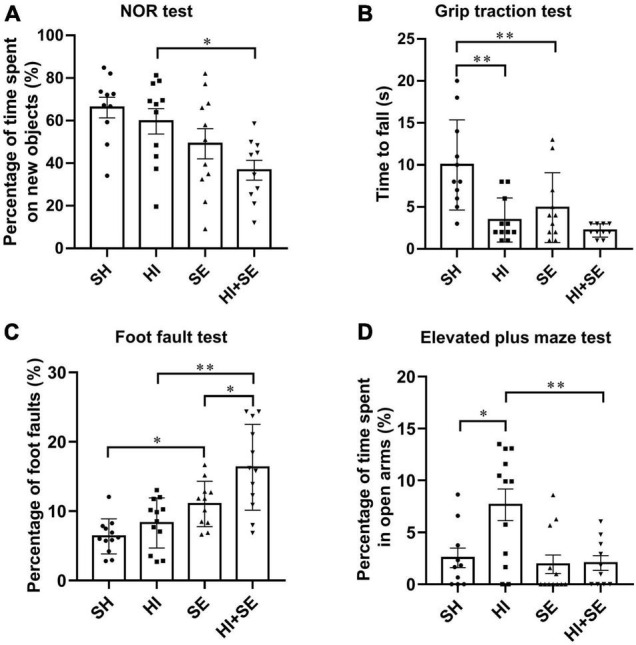
Behavioral test results at P40–44. Novel object recognition (NOR) test **(A)**, grip traction test **(B)**, foot fault test **(C)**, and elevated plus maze test **(D)** in female offspring at P40–44 (*n* = 10). Results are expressed as mean ± SEM. *P < 0.05, **P < 0.01, analyzed by two-way ANOVA followed by *post hoc* Turkey tests. SH, from sham exposed dams with sham surgery; HI, hypoxic-ischemic injury; SE, from smoke exposed dams with sham surgery; HI + SE, from smoke exposed dams with hypoxic-ischemic injury.

In the Novel Object Recognition test, HI injury alone did not affect the short-term memory in either control or SE offspring (Figure 1A). However, maternal SE led to decreased time spent with the novel object in the offspring with HI injury (*P* < 0.05, *F* = 11.78, HI + SE vs. HI, Figure 1A).

In the grip traction test, maternal SE reduced the grip strength (*P* < 0.01, *F* = 7.871, SE vs. SH, Figure 1B). HI injury significantly reduced hanging time in the control offspring (*P* < 0.01, *F* = 16.74, HI vs. SH, Figure 1B). However, there was no additive effect of maternal SE and HI on the grip strength (HI + SE vs. HI and HI + SE vs. SE, Figure 1B).

In the foot faults test, SE offspring made more mistakes than the SH mice (*P* < 0.05, *F* = 27.47, Figure 1C). After HI injury, both control and SE offspring made more mistakes than their uninjured littermates (*P* < 0.05, *F* = 8.834, HI effect on both pairs). In addition, offspring from SE dams made even more mistakes compared with the control offspring with HI (*P* < 0.01, *F* = 27.47, HI + SE vs. HI, Figure 1C).

In the elevated plus maze test, mice with HI injury spent more time in open arms compared with the SH group (*P* < 0.05, *F* = 5.576, HI vs. SH, Figure 1D), which was restored in the HI + SE group (*P* < 0.01, *F* = 7.922, HI + SE vs. HI, Figure 1D).

### Maternal Smoke Exposure and Hypoxia-Ischemic Injury Reduced Neuronal Density and Synaptic Markers

**FIGURE 2:**
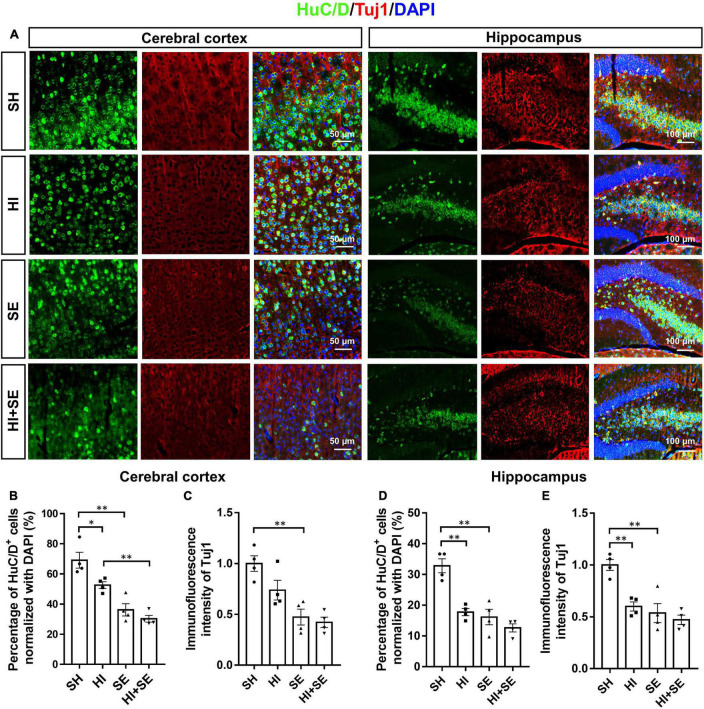
Maternal SE exaggerated HI injury induced neuronal loss. Representative images of immunofluorescence staining of HuC/D and Tuj1 in the cerebral cortex and hippocampus **(A)**. Percentage of HuC/D immunoreactive (HuC/D^+^) cells **(B,D)** and immunofluorescence intensity of Tuj1 **(C,E)** in the cerebral cortex and hippocampus. Results are presented as mean ± SEM. *P < 0.05, **P < 0.01, *n* = 4, analyzed by two-way ANOVA followed by *post hoc* Turkey tests. SH, from sham exposed dams with sham surgery; HI, hypoxic-ischemic injury; SE, from smoke exposed dams with sham surgery; HI + SE, from smoke exposed dams with hypoxic-ischemic injury.

To determine the neuronal loss, double staining of neuronal cell makers Tuj1 and HuC/D were used to show the neuronal density (Figure 2A). Tuj1 is a member of beta tubulin protein family in neurons, which can show the density of nerve fibers. HuC/D are neuronal RNA-binding proteins located in the cell body and can show the number of neuronal cells (Desmet et al., 2014; Huang et al., 2021). Double staining of these two neuronal markers can reveal the loss of both nerve fibers and the number of neuronal cells. In the cerebral cortex, the SE offspring had only half of the number of HuC/D immunoreactive cells and Tuj1 level than the controls (*P* < 0.05, *F* = 56.34 and 29.74, SE vs. SH, Figures 2B,C). A similar pattern was observed for both pan-neuronal markers in the hippocampal region (*P* < 0.01, *F* = 32.65 and 23.29, SE vs. SH, Figures 2D,E). HI injury reduced HuC/D positive cells and Tuj1 levels in the cerebral cortex (both *P* < 0.01, *F* = 56.34 and 29.74, HI vs. SH, Figures 2B,C) and hippocampus (both *P* < 0.01, *F* = 32.65 and 23.29, HI vs. SH, Figures 2D,E) only in the offspring from Sham exposed dams. There were no additive effects of maternal SE and HI on either marker.

**FIGURE 3:**
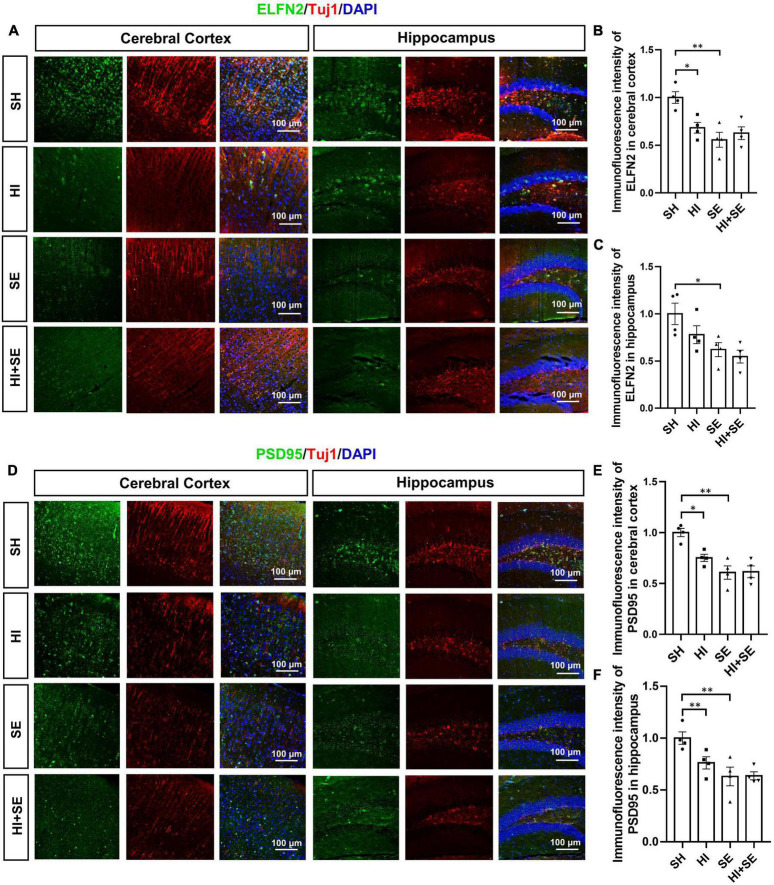
HI injury and maternal SE led to synaptic protein loss. Representative images of ELFN2 and Tuj1 immunofluorescence staining in the cerebral cortex and hippocampus **(A)**. The fluorescence intensity of ELFN2 in the cerebral cortex **(B)** and hippocampus **(C)**. Representative images of PSD95 and Tuj1 immunofluorescence staining in cerebral cortex and hippocampus **(D)**. The fluorescence intensity of PSD95 in the cerebral cortex **(E)** and hippocampus **(F)**. Results are presented as mean ± SEM. *P < 0.05, **P < 0.01, *n* = 4, analyzed by two-way ANOVA followed by *post hoc* Turkey tests. SH, from sham exposed dams with sham surgery; HI, hypoxic-ischemic injury; SE, from smoke exposed dams with sham surgery; HI + SE, from smoke exposed dams with hypoxic-ischemic injury.

To determine the changes in synaptic plasticity, double staining of postsynaptic markers ELFN2 and PSD95 were used. Both ELFN2 and PSD95 are involved in signaling transduction via synapses. Dysfunctions of ELFN2 or PSD95 have been related to cognitive or learning deficits (Fernandez et al., 2017; Matsunaga and Aruga, 2021). Double staining of synaptic proteins and Tuj1 can reveal the correlation between the decreased synaptic proteins and neuronal loss. Maternal SE resulted in fewer ELFN2 and PSD95 in both the cerebral cortex and hippocampus (*P* < 0.05, *F* = 13.5, 11.65, 26.66, and 14.69, respectively; SE vs. SH, Figures 3A–F). HI injury reduced the levels of ELFN2 in the cortex (*P* < 0.05, *F* = 6.406; HI vs. SH, Figure 3B) and PSD95 in both cortex and hippocampus (*P* < 0.05 and *P* < 0.01, *F* = 5.546 and 3.277, respectively; HI vs. SH, Figures 3D–F). There were no additive effects of maternal SE and HI on either marker.

### Maternal Smoke Exposure, but Not Hypoxia-Ischemic Injury, Increased Apoptosis

**FIGURE 4:**
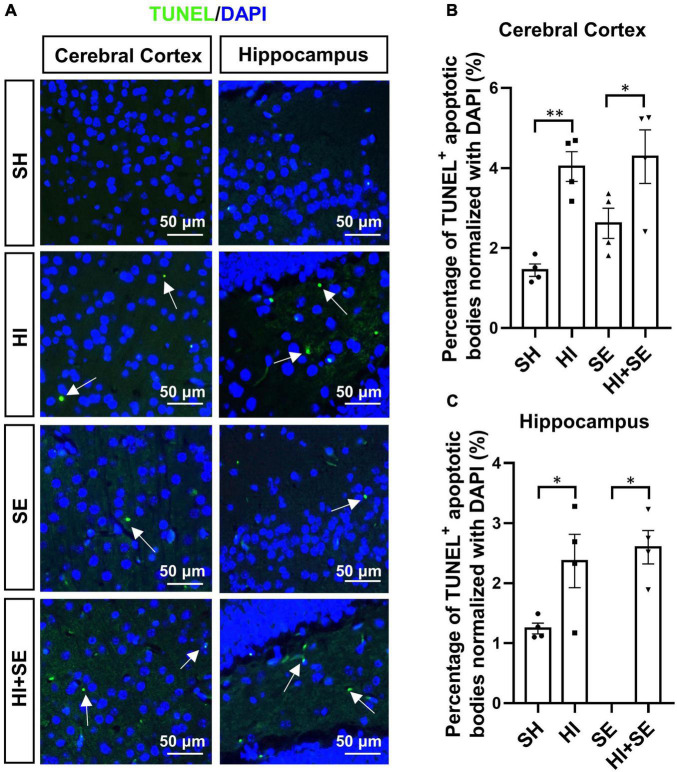
HI injury increased cell apoptosis in the brain. Representative images of TUNEL staining in cerebral cortex and hippocampus (arrows: TUNEL positive apoptotic body) **(A)**. The percentage of TUNEL immunoreactive (TUNEL^+^) apoptotic bodies in the cerebral cortex **(B)** and hippocampus **(C)**. Results are presented as mean ± SEM. *P < 0.05, **P < 0.01, *n* = 4, analyzed by two-way ANOVA followed by *post hoc* Turkey tests. SH, from sham exposed dams with sham surgery; HI, hypoxic-ischemic injury; SE, from smoke exposed dams with sham surgery; HI + SE, from smoke exposed dams with hypoxic-ischemic injury.

The apoptosis was determined by TUNEL assay which reflects the genomic DNA breaks during apoptosis (Figure 4A). Maternal SE increased cell death by 91% in the cerebral cortex and 28% in the hippocampus compared with SH offspring, albeit without statistical significance (Figures 4B,C). HI injury increased the numbers of apoptotic cells to similar levels in both cerebral cortex and hippocampus in control and SE offspring (*P* < 0.05, *F* = 24.08 and 13.58, HI vs. SH and HI + SE vs. SE, Figures 4B,C). There were no additive effects of maternal SE and HI injury on apoptosis marker reflected by TUNEL positive cell numbers (Figures 4B,C).

### Maternal Smoke Exposure Exacerbated Glial Activation Due to Hypoxia-Ischemic Injury

**FIGURE 5:**
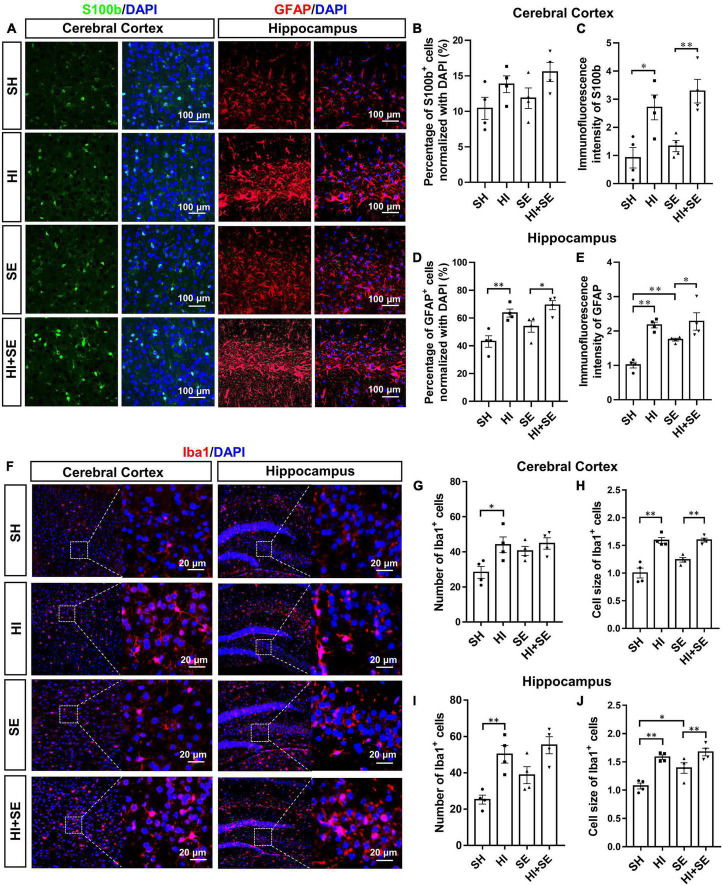
Glial cell activation induced by HI injury and maternal SE. Representative images of immunofluorescence staining of S100b in the cerebral cortex and GFAP in the hippocampus **(A)**. The percentage of S100b immunoreactive (S100b^+^) cells **(B)** and immunofluorescence intensity of S100b **(C)** in the cerebral cortex. The percentage of GFAP immunoreactive (GFAP^+^) cells **(D)** and immunofluorescence intensity of GFAP **(E)** in the hippocampus. Representative images of immunofluorescence staining of Iba1 in cerebral cortex and hippocampus **(F)**. The number and cell size of Iba1 immunoreactive (Iba1^+^) cells in the cerebral cortex **(G,H)** and hippocampus **(I,J)**. Results are presented as mean ± SEM. *P < 0.05, **P < 0.01, *n* = 4, analyzed by two-way ANOVA followed by *post hoc* Turkey tests. SH, from sham exposed dams with sham surgery; HI, hypoxic-ischemic injury; SE, from smoke exposed dams with sham surgery; HI + SE, from smoke exposed dams with hypoxic-ischemic injury.

To examine the activation of astrocytes, double staining of S100b and GFAP were performed. Astrocyte activation is characterized by increased levels of the astrocytic markers S100b and GFAP (Brozzi et al., 2009; Lananna et al., 2018). S100b and GFAP levels vary in different encephalic regions, thus S100b and GFAP were used to quantify the activation of astrocytes in cortical and hippocampal areas, respectively. Maternal SE did not affect astroglial marker S100b staining in the cortex (Figures 5A–C). However, it increased GFAP immunofluorescence intensity in the hippocampus (*P* < 0.01, *F* = 5.138; SE vs. SH, Figure 5E). HI injury increased S100b immunofluorescence intensity in the cortex in both controls (*P* < 0.05, *F* = 6.51; HI vs. SH, Figure 5C) and SE offspring (*P* < 0.01, *F* = 6.51; HI + SE vs. SE, Figure 5C). It also increased astrocyte activation in the hippocampus with more GFAP^+^ cell number and higher immunofluorescence intensity in both control and SE offspring (*P* < 0.01 HI vs. SH, *F* = 24.61 and 25.85; *P* < 0.05 HI + SE vs. SE, Figures 5D,E).

A specific marker of microglia, Iba1 was used to reveal the numeric and morphologic changes of activated microglia by maternal SE and HI injury (Hendrickx et al., 2017). Maternal SE did not significantly change Iba1 immunoreactive microglial cell number and size in the cortex (Figures 5F–H), but increased microglial cell size in the hippocampus (*P* < 0.05, *F* = 9.362; SE vs. SH, Figure 5J). HI injury significantly increased microglial cell numbers in both the cortex (*P* < 0.05, *F* = 8.184; HI vs. SH, Figure 5G) and hippocampus (*P* < 0.05, *F* = 23.46; HI vs. SH, Figure 5I) in the control offspring, and increased microglial cell size in the cortex and hippocampus in both control and SE offspring (*P* < 0.01, *F* = 66.11 and 35.59; HI vs. SH and HI + SE vs. SE, Figures 5H,J). There were no additive effects of maternal SE and HI.

### Maternal Smoke Exposure Exacerbated Oxidative Stress and Inflammatory Response Due to Hypoxia-Ischemic Injury in the Cerebral Cortex

**FIGURE 6:**
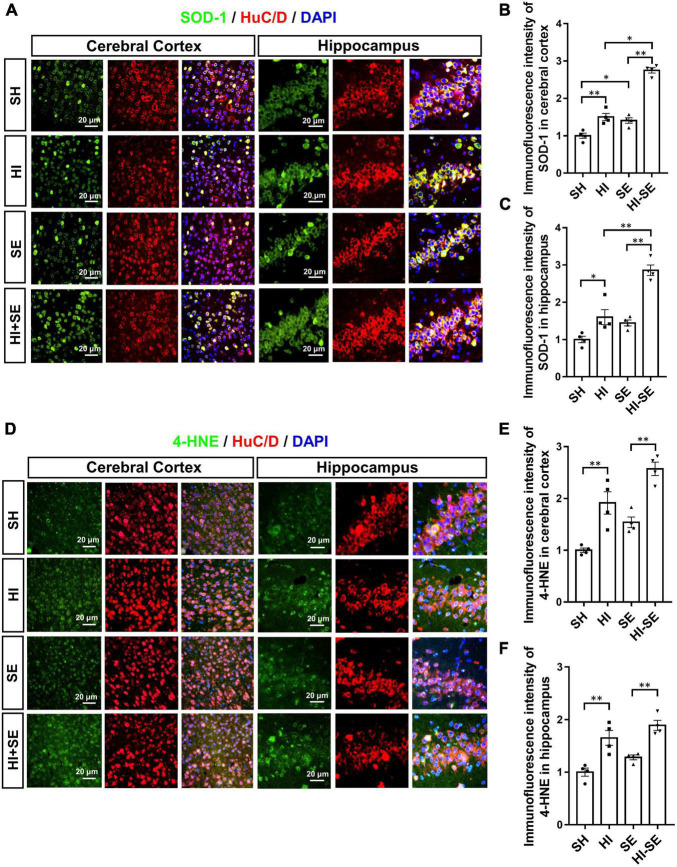
Maternal SE enhanced oxidative stress caused by HI injury. Representative images of SOD-1 and HuC/D immunofluorescence staining in the cerebral cortex and the hippocampus **(A)**. The immunofluorescence intensity of SOD-1 in the cerebral cortex **(B)** and hippocampus **(C)**. Representative images of 4-HNE and HuC/D immunofluorescence staining in the cerebral cortex and the hippocampus **(D)**. The immunofluorescence intensity of 4-HNE in the cerebral cortex **(E)** and the hippocampus **(F)**. Results are presented as mean ± SEM. *P < 0.05, **P < 0.01, *n* = 4, analyzed by two-way ANOVA followed by *post hoc* Turkey tests. SH, from sham exposed dams with sham surgery; HI, hypoxic-ischemic injury; SE, from smoke exposed dams with sham surgery; HI + SE, from smoke exposed dams with hypoxic-ischemic injury.

SOD-1 is the primary antioxidant enzyme responsible for the removal of the superoxide anion during oxidative stress (Kwiatkowska et al., 2017); while, 4−hydroxynonene (4−HNE) is a stable end product of lipid peroxidation whose the level can be increased by hypoxia (Zhao et al., 2019). Double staining of SOD-1 or 4-HNE together with Tuj1 can reflect the level of oxidative stress in the neuronal cells. Maternal SE led to higher levels of SOD-1 in the cortex (*P* < 0.05, *F* = 114.2; SE vs. SH, Figures 6A,B), however, only marginally increased the levels of SOD-1 and 4-HNE in the hippocampus (SE vs. SH, Figures 6C,D,F). HI injury significantly increased SOD-1and 4-HNE levels in both cortical and hippocampal neurons in the control and SE offspring (*P* < 0.05, *F* = 141.7, 54.29, 49.64, and 42.55; HI vs. SH, HI + SE vs. SE, Figures 6B,C,E,F). In addition, SE offspring had higher levels of SOD-1 after HI injury (*P* < 0.05 in the cortex, *P* < 0.01 in the hippocampus, *F* = 114.2 and 38.73; HI + SE vs. HI, Figures 6B,C).

**FIGURE 7:**
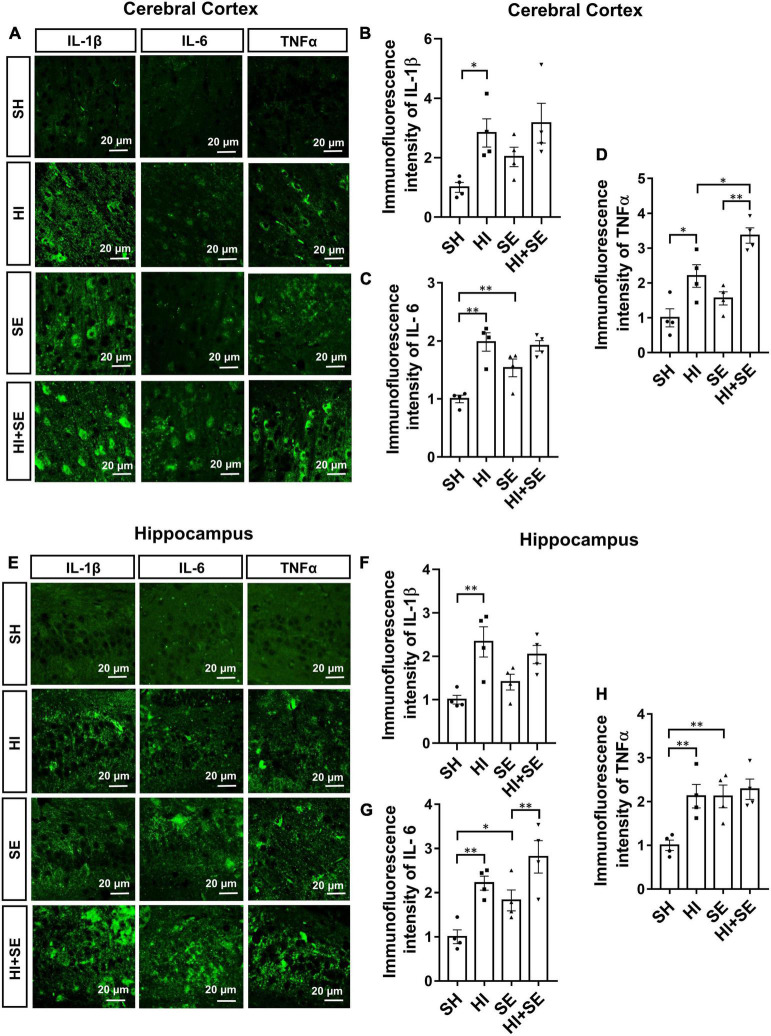
Inflammatory response induced by HI injury and maternal SE. Representative images of immunofluorescence staining of inflammatory cytokines IL-1β, IL-6, and TNFα in the cerebral cortex **(A)** and the hippocampus **(E)**. The immunofluorescence intensity of IL-1β **(B,F)**, IL-6 **(C,G)**, and TNFα **(D,H)** in the cerebral cortex and hippocampus. Results are presented as mean ± SEM. *P < 0.05, **P < 0.01, *n* = 4, analyzed by two-way ANOVA followed by *post hoc* Turkey tests. SH, from sham exposed dams with sham surgery; HI, hypoxic-ischemic injury; SE, from smoke exposed dams with sham surgery; HI + SE, from smoke exposed dams with hypoxic-ischemic injury.

To examine the inflammatory response, the levels of different pro-inflammatory cytokines typically released by the microglia were measured, including IL-1β, IL-6, and TNFα. Maternal SE increased IL-6 levels in the cortex and hippocampus (both *P* < 0.01, *F* = 3.613 and 8.399, SE vs. SH, Figures 7A,C,E,G), TNFα in the hippocampus (*P* < 0.05, *F* = 7.844; SE vs. SH, Figure 7H), without affecting IL-1β levels in either region (Figures 7B,F). HI injury significantly increased the levels of IL-1β, IL-6, and TNFα in both the cerebral cortex and hippocampus of the control offspring (*P* < 0.05, *F* = 10.89, 30.27, 34.88, 18.56, 20.19, and 7.999, HI vs. SH, Figures 7B–D,F–H); however, it only increased TNFα in the cortex (*P* < 0.01, *F* = 34.88, HI + SE vs. SE, Figure 7D) and IL-6 level in the hippocampus of the SE offspring (*P* < 0.01, *F* = 20.19, HI + SE vs. SE, Figure 7G). Additionally, TNFα level in the cortex of the HI + SE mice was also significantly higher than that in the HI group (*P* < 0.05, *F* = 11.37; Figure 7D).

## Discussion

Tobacco use remains a major public health issue, especially first- and second-hand smoking during pregnancy which can significantly compromise the health outcome in the unborn child. In this study, we found that neurobehavioral outcomes in the adolescent female offspring were impaired due to *in utero* SE exposure, which were worsened by postnatal HI insult. Although male offspring were more vulnerable to the impact of maternal SE than females without additional postnatal insult (Chan et al., 2017a), against our hypothesis, the impairments in memory and motor functions of the females reported here were similar to their male littermates published in our previous study (Chan et al., 2017b). Although sex difference was not found in the neurological phenotype, the changes seem to be driven by different mechanisms in females and males.

In this study, female offspring displayed reduced motor function due to maternal SE, which was similar to the male littermates (Chan et al., 2017b). In addition, the short-term memory function of male SE offspring in our previous study (Chan et al., 2017b) and their female littermates in this study was only impaired following HI injury, suggesting non-sexual bias in response to both maternal SE and HI injury. However, the changes in anxiety level were different in SE female offspring from their male littermates (Chan et al., 2017b). While control offspring were more relaxed after HI injury, SE females did not have any change. On the other hand, their male littermates were anxious without injury, which was restored to control level after HI injury (Chan et al., 2017b); whereas in the control female offspring, the HI injury increased their anxiety levels, which was not affected in those from SE dams. In humans, maternal smoking, especially during early pregnancy, is linked to anxiety behaviors in late adolescence, more so in boys than girls (Bing et al., 2001). This effect seems to be nicotine dose-dependent, as shown in a rodent model (Cheeta et al., 2001). While patients with HI injury, such as stroke, often show increased anxiety due to the impact on their living, rodent models showed reduced anxiety days after the stroke (Ruan and Yao, 2020). This is consistent with the control offspring with HI injury, suggesting anxiety in humans may be psychological rather than physiology. Nevertheless, SE offspring lost such “benefit” on mental health, which may be linked to the pathological changes in their brain.

Neuronal cell death and synaptic loss are the major events of perinatal HI injury, leading to neurobehavioral changes in the control offspring with HI injury. The increased apoptosis and loss of variable neurons and synapses interweaved with inflammatory response and oxidative stress (Vannucci and Hagberg, 2004). ELFN2 is a postsynaptic cell adhesion molecule with essential roles in both organizing and tuning glutamatergic neurotransmission in the brain. As ELFN2 mainly inhibits glutamate release, Elfn2 knockout mice display multiple neuropsychiatric phenotypes, including increased seizure susceptibility, hyperactivity, and anxiety/compulsivity (Dunn et al., 2019). ELFN2 was significantly reduced in the cerebral cortex by maternal SE and HI injury and decreased in hippocampal regions by maternal SE. Such changes were somewhat in line with reduced neural fiber density and cell numbers. Therefore, we postulate that the reduced protein level was due to fewer neurons which may not correlate to reduced glutamate release. Thus, HI injury in control offspring reduced anxiety levels, which was not seen in SE offspring with HI injury. The changes in anxiety level in females are opposite to our previous finding in males, where maternal SE caused higher levels of anxiety which was normalized by postnatal HI injury (Chan et al., 2017b). Thus, this sex difference in anxiety was also found in humans (Altemus et al., 2014; Maeng and Milad, 2015). PSD95, another postsynaptic molecule, is associated with the maturation of glutamatergic synapses and contributes to synapse stabilization and plasticity (El-Husseini et al., 2000). Previous studies showed reduced PSD95 levels by HI insult and cigarette smoking in neonatal mice (Ho et al., 2012; Shao et al., 2017), which may explain reduced memory function in SE offspring with HI injury. Interestingly, the short-term memory was not significantly changed in the control offspring with HI injury in this study. A previous study showed reduced memory function at P32, which was recovered at P46 in mice with neonatal HI injury (Reinboth et al., 2016), suggesting that neural plasticity is more resilient in injured neonatal brains without maternal SE.

The nicotine from the cigarette smoke inhaled by the smoking mothers has been shown to induce placental infarction and microinfarction, which can reduce blood and oxygen supply to the fetus leading to brain underdevelopment (Kaminsky et al., 2007; Fatemi et al., 2009). Nicotine can also be transmitted through the placenta and lactation to directly affect early brain development, especially the neuronal cell maturation and synapse formation (Aramakis et al., 2000; Chan et al., 2016b). Thus, in the current study, neuron cell density and synaptic protein levels in the SE offspring were nearly half of the control offspring, even without any postnatal injury. Such changes well supported their impaired motor functions. This phenomenon also reflects the redundancy of neurogenesis during development to allow later re-adjustment by environmental cues (Pfisterer and Khodosevich, 2017; Hiratani and Fukai, 2018). In this study, although both maternal SE and HI resulted in decreased neuronal density and synaptic protein levels (especially in the hippocampus), they may be driven by different machines, e.g., increased apoptosis due to HI injury and reduced neurogenesis by maternal SE (Aramakis et al., 2000; Chu et al., 2019). However, the reduced neurogenesis caused by maternal SE may have reached the minimum number of neurons required for survival; therefore, neuronal number and synaptic protein were preserved in response to additional HI injury. However, adverse micro-environment may still affect neural function, such as increased oxidative stress and inflammation, demonstrated by exaggerated impairment of short-term memory function in SE offspring with Hi injury.

Oxidative stress also plays an important role in the pathogenesis and progression of HI brain injury. Following the HI event, the inflammatory response can induce significant oxidative stress by the production of excess ROS, which can future trigger the release of more free radicals and cause calcium overloading, leading to excitotoxicity, ionic imbalance, inflammation, apoptosis, and necrosis (Doyle et al., 2008). Our previous study has shown that female offspring from SE dams were better at producing endogenous antioxidants than male littermates; therefore, when there is no additional postnatal insult, their brains seem to be protected from excessive apoptosis observed in their male littermates (Chan et al., 2016a,b). As a lipid oxidation product, increased 4-HNE is closely related to apoptosis and the pathogenesis of several neurodegenerative diseases (Negre-Salvayre et al., 2010). On the other hand, SOD-1 is protective for HI conditions, such as stroke (Spranger et al., 1997; Pagan et al., 2006). In the current study, HI increased the levels of both 4-HNE and SOD-1 in offspring from both control and SE dams, with SOD-1 increasing even higher in SE offspring in both cerebral cortex and hippocampal regions. These results suggest an adaptive increase in the antioxidant SOD-1 due to oxidative stress reflected by 4-HNE level, which was, however, unsuccessful in protecting affected brain regions regardless of maternal status.

In line with increased oxidative stress, we also observed a heightened inflammatory response in SE offspring with HI injury. Long-term exposure to cigarette smoke caused a significant increase in brain glial cells, including microglia and astrocyte (Khanna et al., 2013). Such effects also occurred in the offspring’s brain if dams were exposed to cigarette smoke during pregnancy. After a HI attack, resident immune cells in the brain are activated by the accumulation of free radicals and then release pro-inflammatory mediators (Jin et al., 2010). While the supporting glia astrocytes were increased aiming to protect the vulnerable neurons in such toxic micro-environment, microglia, the resident immune cell in the brain, also respond vigorously to produce excessive inflammatory cytokines, such as IL-1β, IL-6, and TNFα (Jellema et al., 2013). The prolonged response can also make astrocytes secrete pro-inflammatory cytokines (Tuttolomondo et al., 2008). A rapid increase in the levels of these cytokines leads to direct neural injuries, inducing apoptosis and attracting circulation macrophages to the ischemic site (Favrais et al., 2011; Girard et al., 2012; Green et al., 2012). Here, we did not see an additive effect of maternal SE and HI injury on glial cell numbers, but saw an additive effect on cytokine level increase in the cortex in line with the functional motor deficit. We cannot directly compare this change in female offspring with their male littermates published previously, as the latter was on mRNA expression (Chan et al., 2017b). We can only conclude a similar trend of increased brain inflammatory response by both maternal SE and HI injury between male and female offspring. The combined effects of HI injury and maternal SE on inflammation were only marked in females, correlating with the adaptive response of SOD-1. Whether this will lead to long-term neurocognitive deficits beyond the adolescent stage requires further investigation (Reinboth et al., 2016).

## Conclusion

In this study, we demonstrated that maternal SE worsened the neurological outcomes due to HI injury in adolescent female offspring, most likely driven by exaggerated oxidative stress and inflammatory response. Our study also reinforces the importance of avoiding direct and second-hand smoking during pregnancy to protect the brain health of the unborn child. Future studies can follow up such offspring into early and late adulthood to determine whether these neurological events can lead to early onset neurodegeneration.

## Data Availability Statement

The raw data supporting the conclusions of this article will be made available by the authors on request.

## Ethics Statement

The animal study was reviewed and approved by the Animal Care and Ethics Committee at the University of Technology Sydney (ACEC# 2014-029).

## Author Contributions

TH: experiments, visualization, investigation, and writing the first draft. XH, HL, JQ, NW, YX, YZ, XX, and RL: visualization and investigation. DL: supervision and editing. YLC: experiments and writing. BGO: methodology and writing. CY and HC: conceptualization, methodology, supervision, and writing. All authors contributed to the article and approved the submitted version.

## Conflict of Interest

The authors declare that the research was conducted in the absence of any commercial or financial relationships that could be construed as a potential conflict of interest.

## Publisher’s Note

All claims expressed in this article are solely those of the authors and do not necessarily represent those of their affiliated organizations, or those of the publisher, the editors and the reviewers. Any product that may be evaluated in this article, or claim that may be made by its manufacturer, is not guaranteed or endorsed by the publisher.
